# Supplementary material for: Recurrent pregnancy loss is associated with a pro-senescent decidual response during the peri-implantation window
Source: Commun Biol. 2020 Jan 21;3:37. doi: 10.1038/s42003-020-0763-1 (PMC6972755; doi:10.1038/s42003-020-0763-1)
Supplement: Supplementary file 1 — Supplementary Information [file 42003_2020_763_MOESM1_ESM.docx]

**Supplementary Figures**


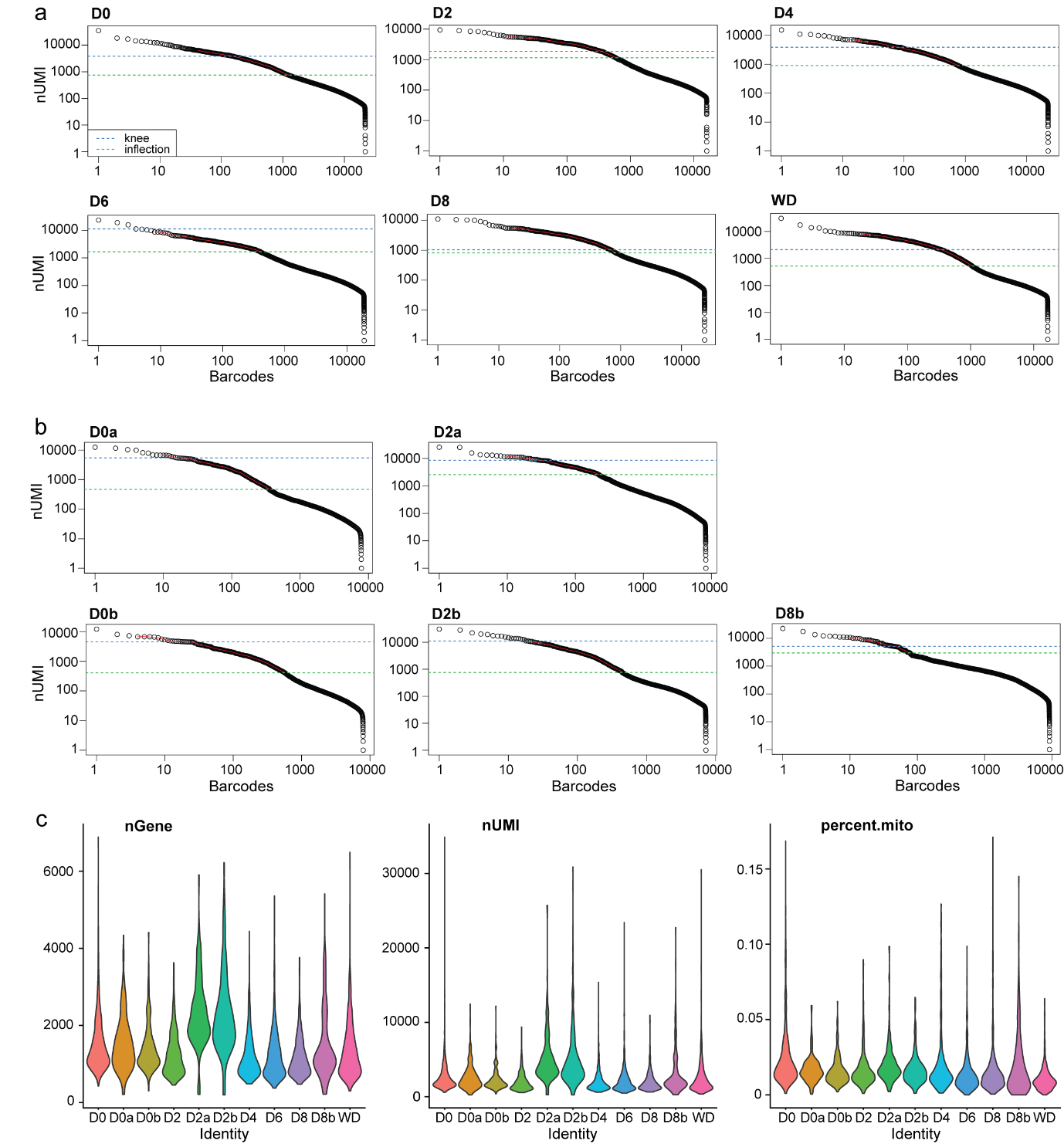


**Supplementary Figure 1. Quality control of timecouse scRNA-seq libraries.** Cumulative read distribution for main (**a**) and additional (**b**) timecourse samples. Knee and inflection points calculated with the DropUtils R package are presented as blue and green dashed lines, respectively. **c**, Violin plots showing distribution of number of genes (nGene), number of transcripts (nUMI) and percent of mitochondrial reads (percent.mito) per timecourse samples**.**

**
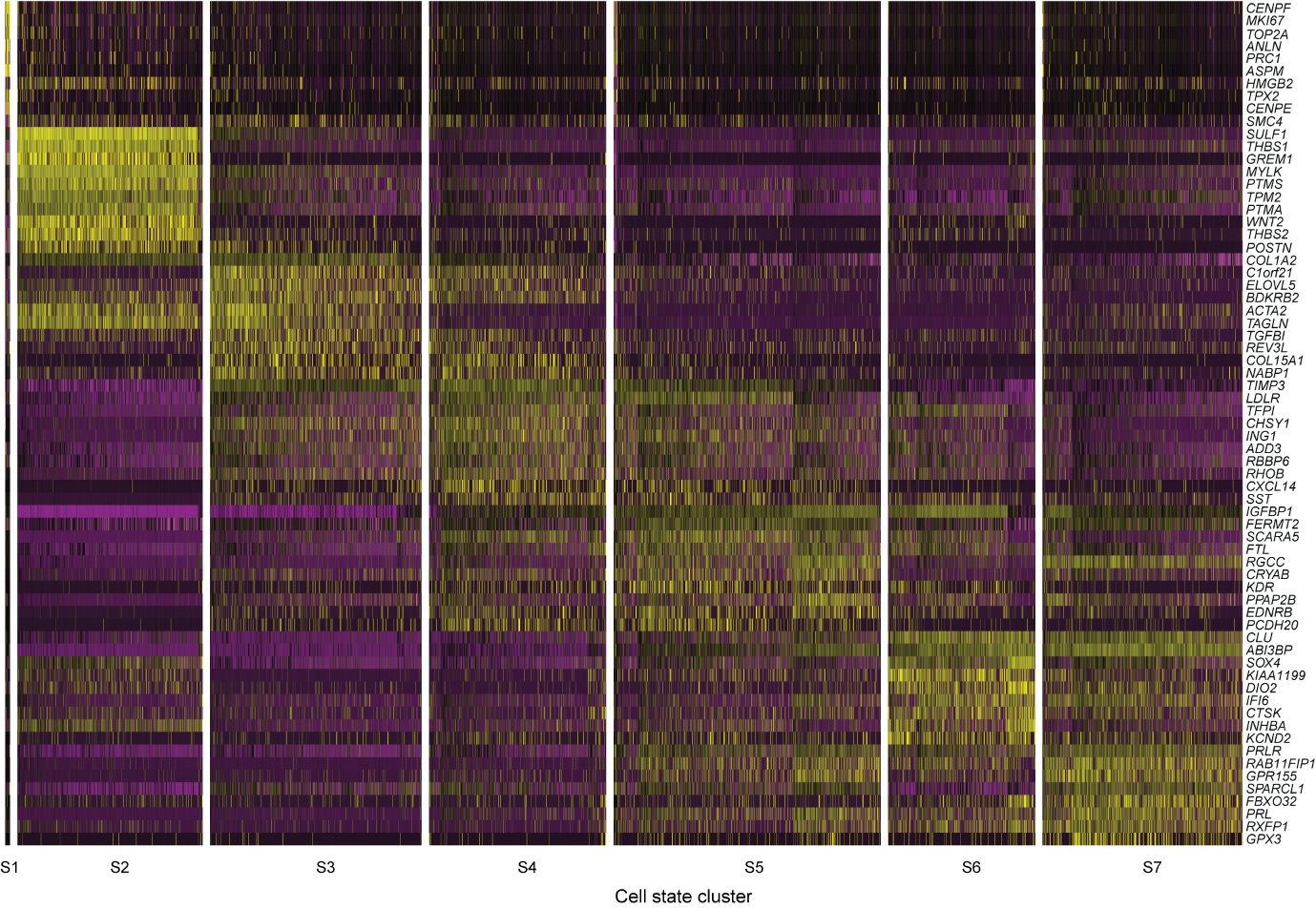
**

**Supplementary Figure 2. Heatmap of the top 10 marker genes for each of the 7 cell states**. Each column represents one of 4,580 EnSC. Expression for each gene is centered to the average expression across cells and scaled by their standard deviation. Yellow, black and purple represents high, medium and low expression, respectively.


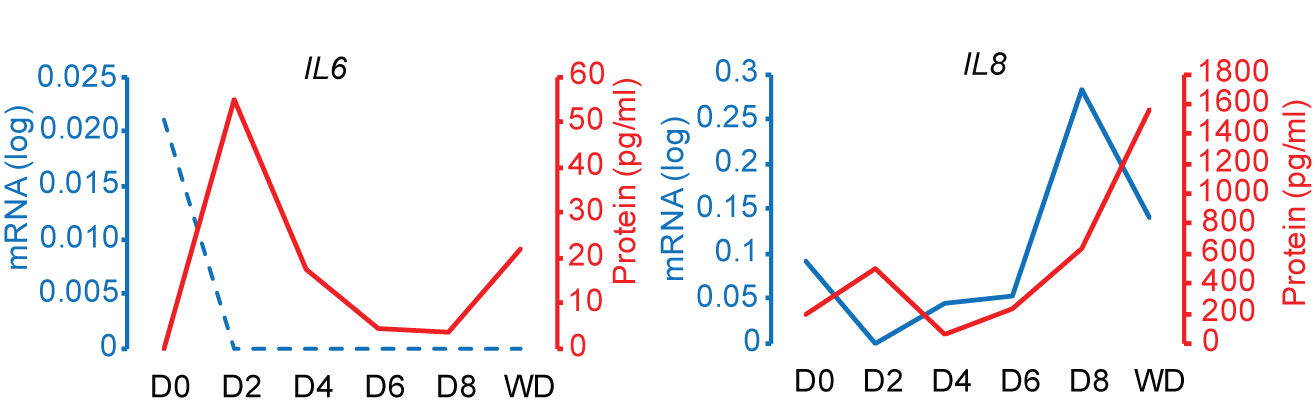


**Supplementary Figure 3. Transient IL-6 and IL-8 secretion during the initial decidual phase.** Temporal profiles of *IL6* and *IL8* transcript levels (blue line) and corresponding secreted levels across the decidual pathway (D0-D8) and upon withdrawal (WD) of differentiation signals. Note that the initial rise in IL-6 or IL-8 secretion does not coincide with a corresponding increase in mRNA levels. A second rise in IL-8 secretion coincides with the emergence of snDC.


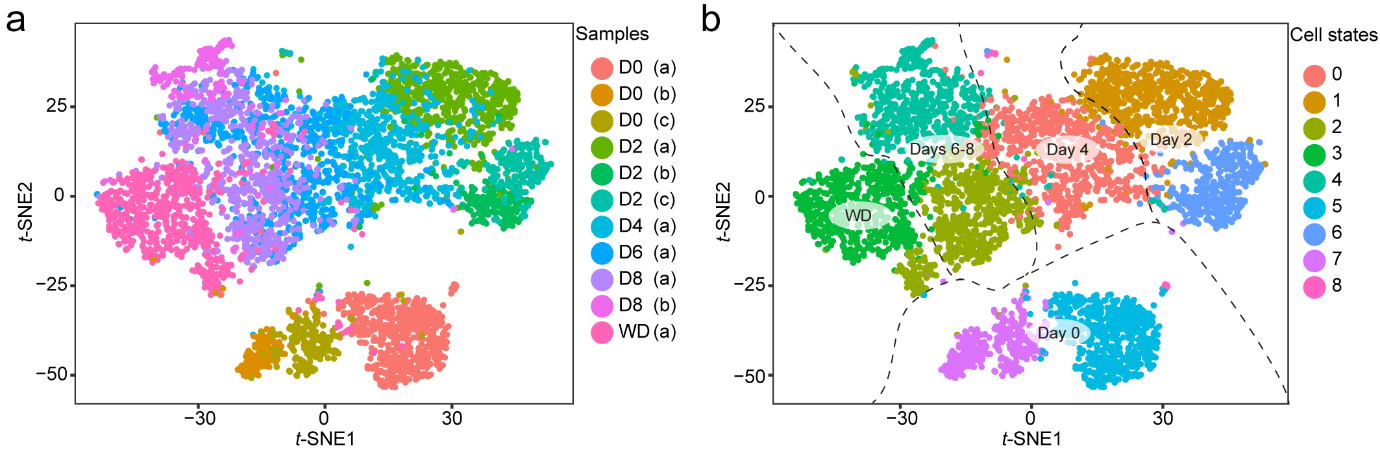


**Supplementary Figure 4. Single-cell analysis of three independent primary cultures shows cells aggregating by transcriptional state**. Three independent primary EnSC cultures (designated a, b, and c) were decidualized for different time-points and then subjected to Drop-seq analysis. Culture ‘a’ represents the full time-course [D0-D8 plus withdrawal (WD)]; culture ‘b’ was decidualized for 2 and 8 days whereas culture ‘c’ was decidualized for 2 days. **a** *t*-SNE plot with cells colour-coded by culture and day of decidualization. **b** *t*-SNE plot with cells colour-coded by cell state. The plot was further annotated to indicate day of decidualization and withdrawal (WD) of differentiation signals.


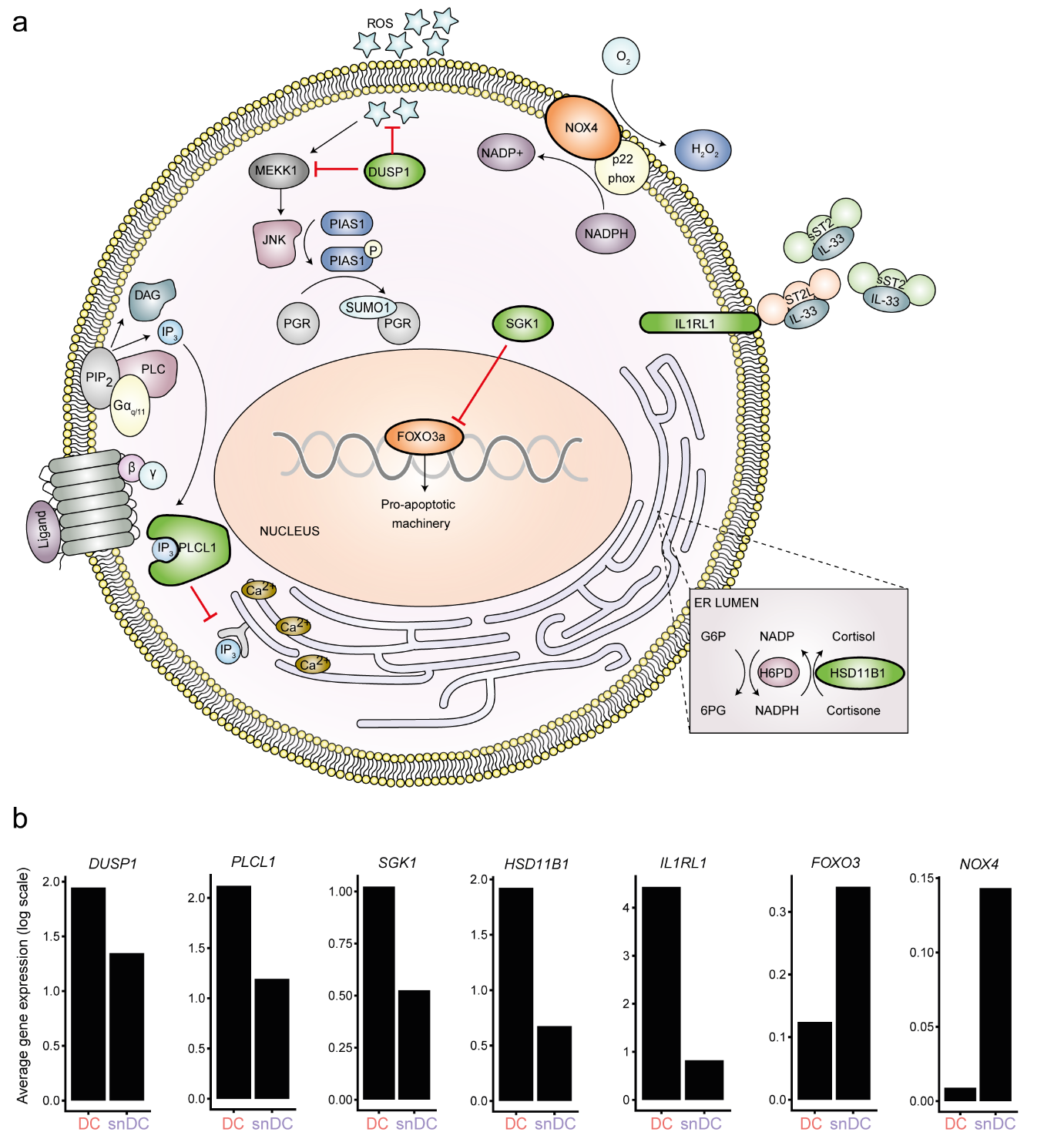


**Supplementary Figure 5. Stress-defence mechanisms in DC**. **a** Multiple mechanisms underpin stress-resistance of DC, including progesterone-dependent induction of dual specificity phosphatase 1 (DUSP1, also known as mitogen-activated protein (MAP) kinase phosphatase-, MKP1), which silences the c-Jun NH-terminal kinase (JNK) stress signaling pathway and blocks stress-dependent sumoylation of numerous targets, including the liganded progesterone receptor (PGR)^1,2^. Progesterone also regulates the expression the serum- and glucocorticoid-inducible kinase SGK1^3^, a kinase that targets and inactivates FOXO3, a key transcription factor involved in oxidative cell death responses in endometrial cells^4^. *PLCL1*, coding phospholipase C like 1 (inactive), is a progesterone-inducible scaffold protein that uncouples phospholipase C activation downstream of Gq-protein-coupled receptors from intracellular Ca^2+^ release by attenuating inositol trisphosphate (IP3) signaling^5^. Progesterone further upregulates 11β-hydroxysteroid dehydrogenase type 1 (encoded by *HSD11B1*)^6,7^, the enzyme that converts inert cortisone into active cortisol, a powerful anti-inflammatory hormone. A highly-induced decidual gene is *IL1RL1*, which encodes the IL-33 transmembrane receptor ST2L as well as the secreted decoy receptor sST2^8^, a potent anti-inflammatory mediator that binds and inactivates IL-33. The main non-mitochondrial source of reactive oxygen species in endometrial stromal cells is NADPH oxidase NOX4^9^. Although initiation of the decidual process requires NOX4 activation^9^, it is also a mediator of cellular senescence^10^. **b** Log-transformed, normalized expression levels for indicated genes in stress-resistant DC (S5) and snDC (S6).


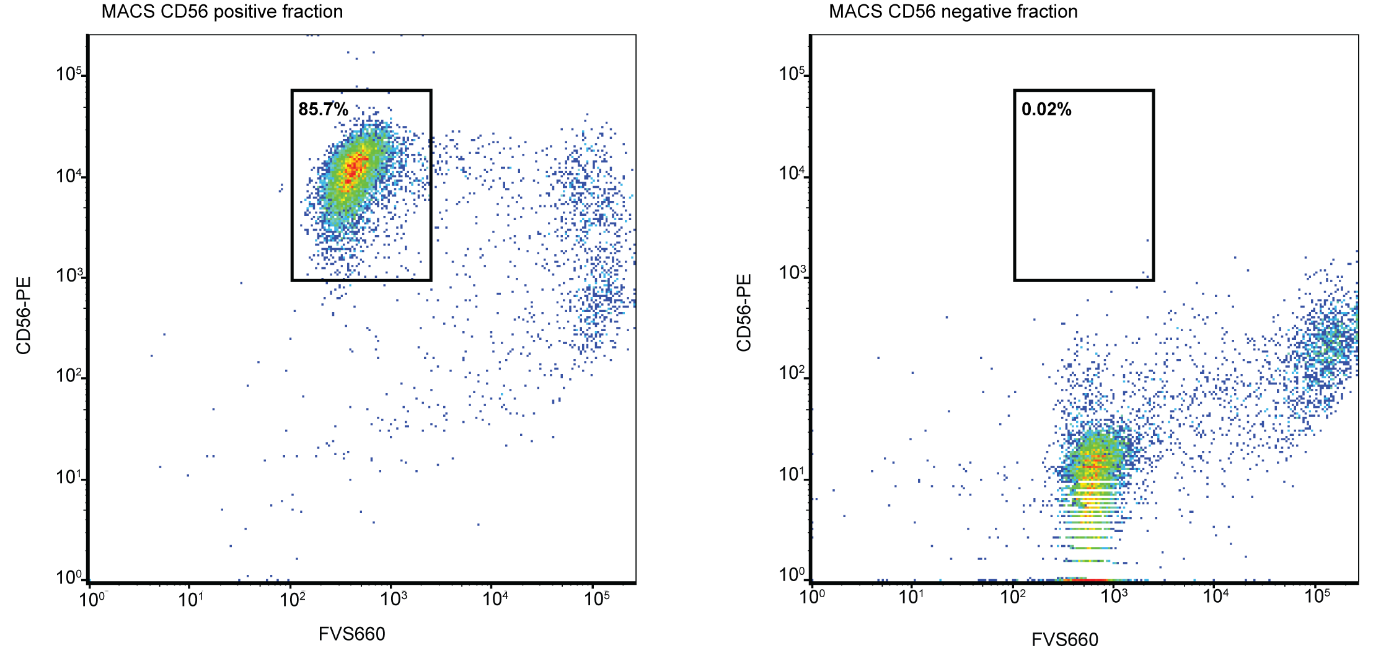


**Supplementary Figure 6. Flow cytometric analysis of CD56^+^ cells following MACS separation.** uNK cells isolated by MACS from the supernatants of 4 independent freshly established EnSC cultures were pooled and then subjected to flow cytometry to confirm enrichment of CD56^+^ cells (CD56-PE) and cell viability (FVS660) in the positive fraction and the absence of CD56^+^ cells in the negative fraction. In the positive fraction, approximately 86% of cells were confirmed to be viable uNK cells.

**
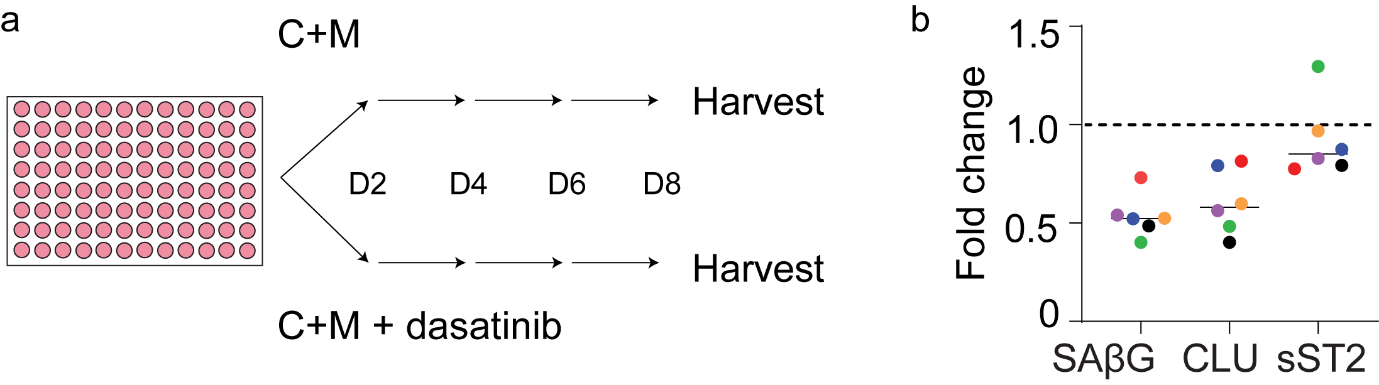
**

**Supplementary Figure 7. Treatment of decidualizing cells with dasatinib mimics uNK co-cultures** Six independent EnSC cultures were decidualized with cAMP and MPA (C+M) for eight days in the presence or absence of the senolytic agent, dasatinib (250 nM). **a** Schematic representation of dasatinib experiments. **b** fold-change in SAβG activity and secretion of clusterin (CLU) and sST2 (encoded by *IL1RL1*) in decidualized cells treated with dasatinib when compared to decidualized cells cultured without dasatinib (dashed line). Individual cultures are indicated by different colours.


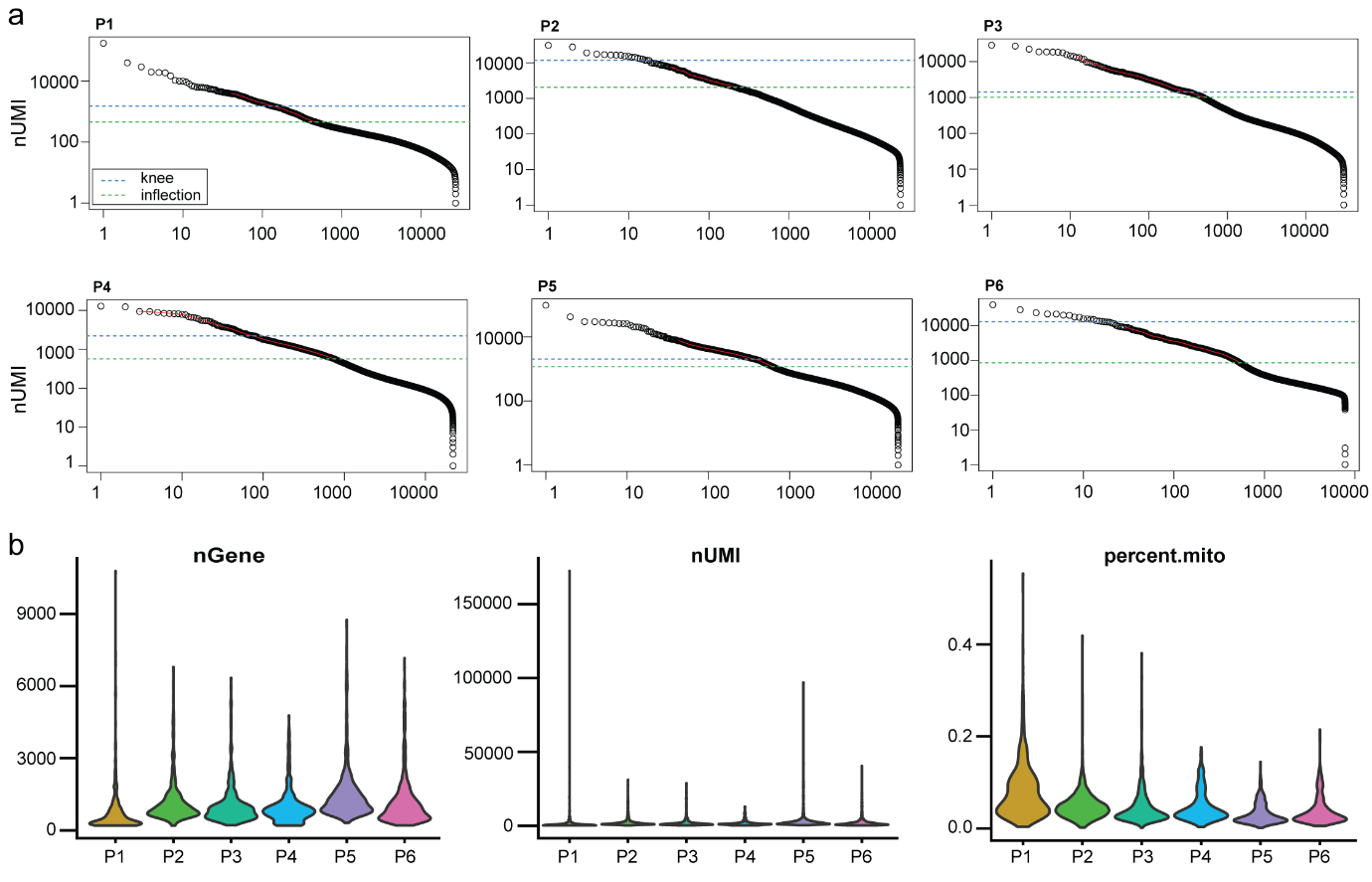


**Supplementary Figure 8. Quality control of biopsy scRNA-seq libraries. a** Cumulative read distribution for biopsy samples. Knee and inflection points calculated with the DropUtils R package are presented as blue and green dashed lines, respectively. **b** Violin plots showing distribution of number of genes (nGene), number of transcripts (nUMI) and percent of mitochondrial reads (percent.mito) per biopsy sample.


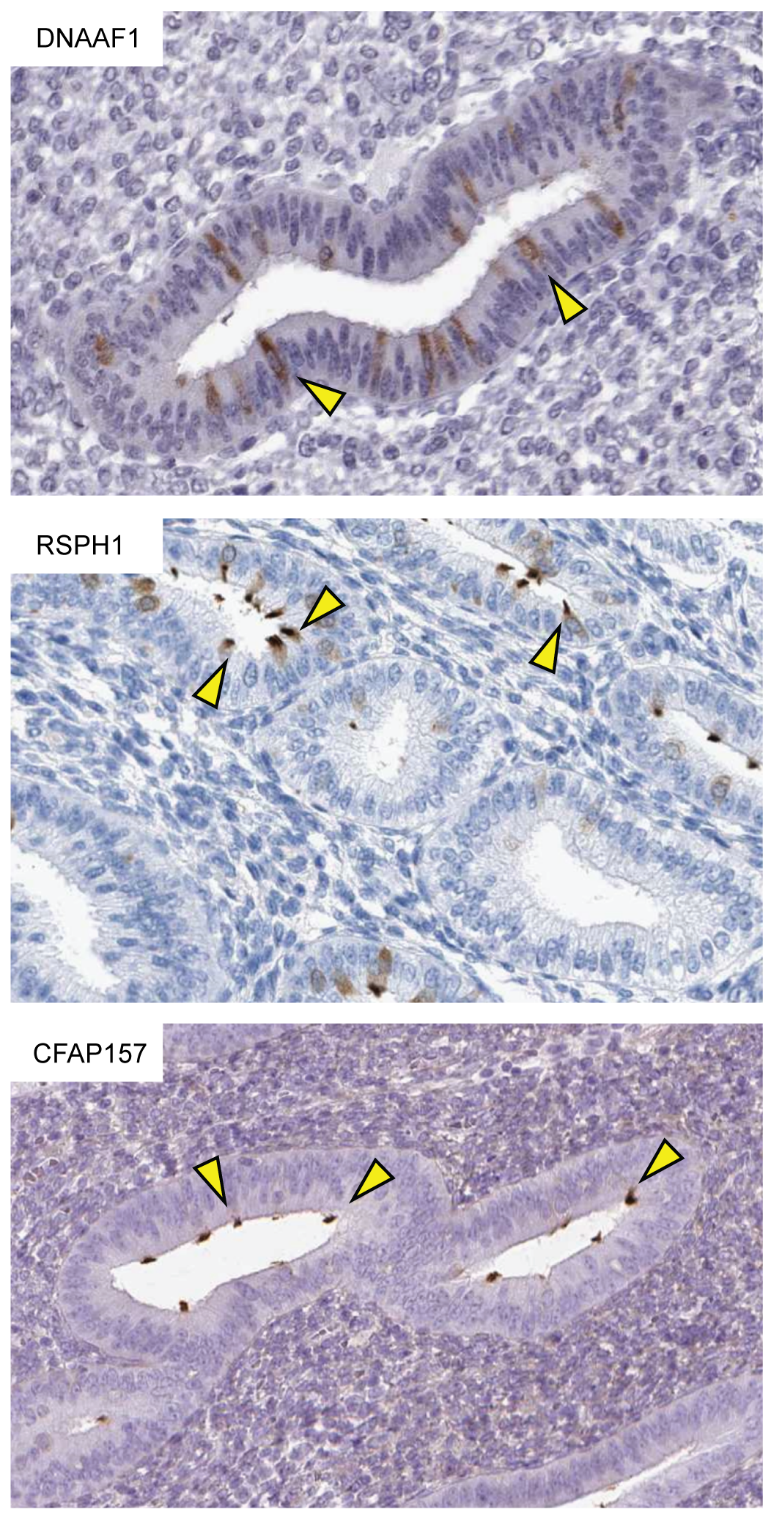


**Supplementary Figure 9. Distribution of ciliated cells in endometrial glandular epithelium.** Arrows indicate the expression of ciliated epithelial markers, dynein axonemal assembly factor 1 (DNAAF1), radial spoke head component 1 (RSPH1), and cilia and flagella associated protein 157 (CFAP157), within the glandular compartment of human endometrium. Images were retrieved from the Human Protein Atlas v18.1 (<https://www.proteinatlas.org/>)^11^.


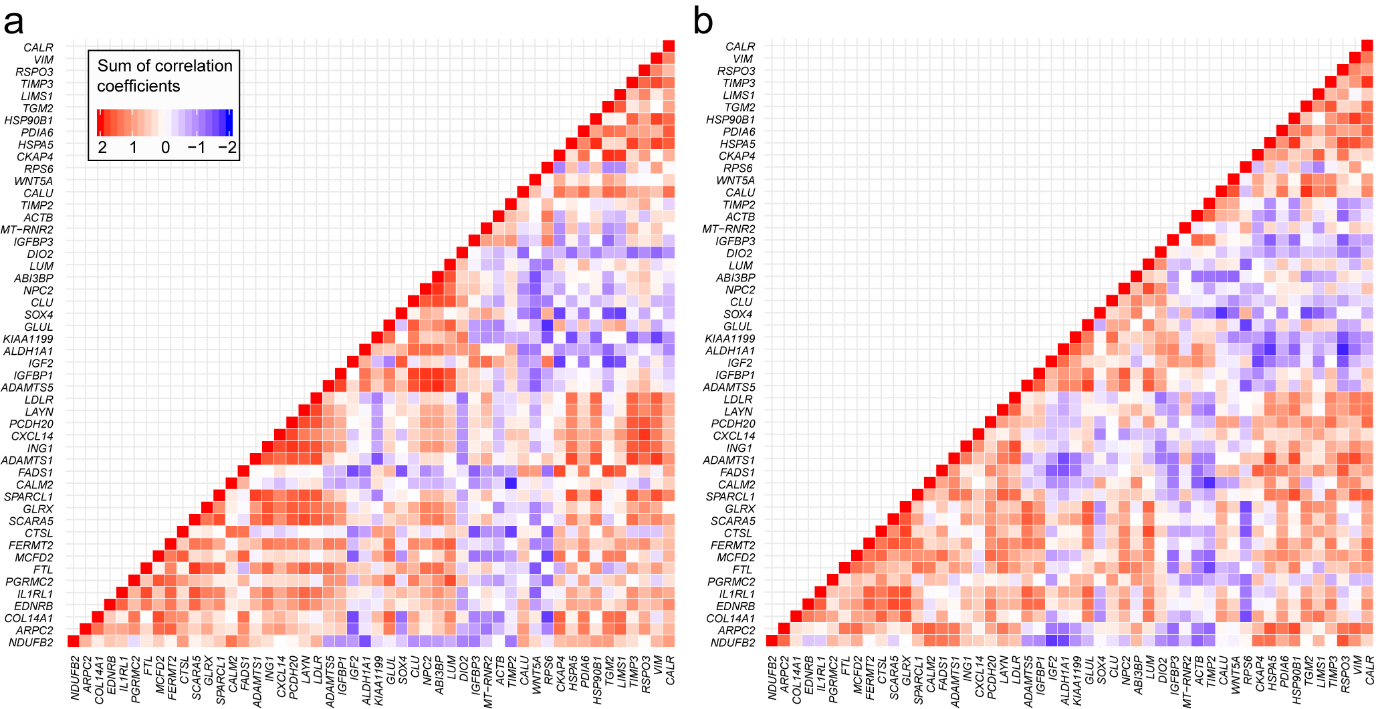


**Supplementary Figure 10. Analysis of *in vitro* branchpoint genes in luteal phase EnSC *in vivo*.** Heatmaps depicting the sum of correlation coefficients of the top 50 gene-gene interactions involved in lineage divergence of decidualizing EnSC *in vitro* compared with *in vivo* expression at LH+8 (**a**) and LH+10 (**b**). The colour key indicates the level of congruency, defined as the sum of correlation coefficients of >1 or <-1 for positively (red) and negatively (blue) co-regulated genes, respectively.


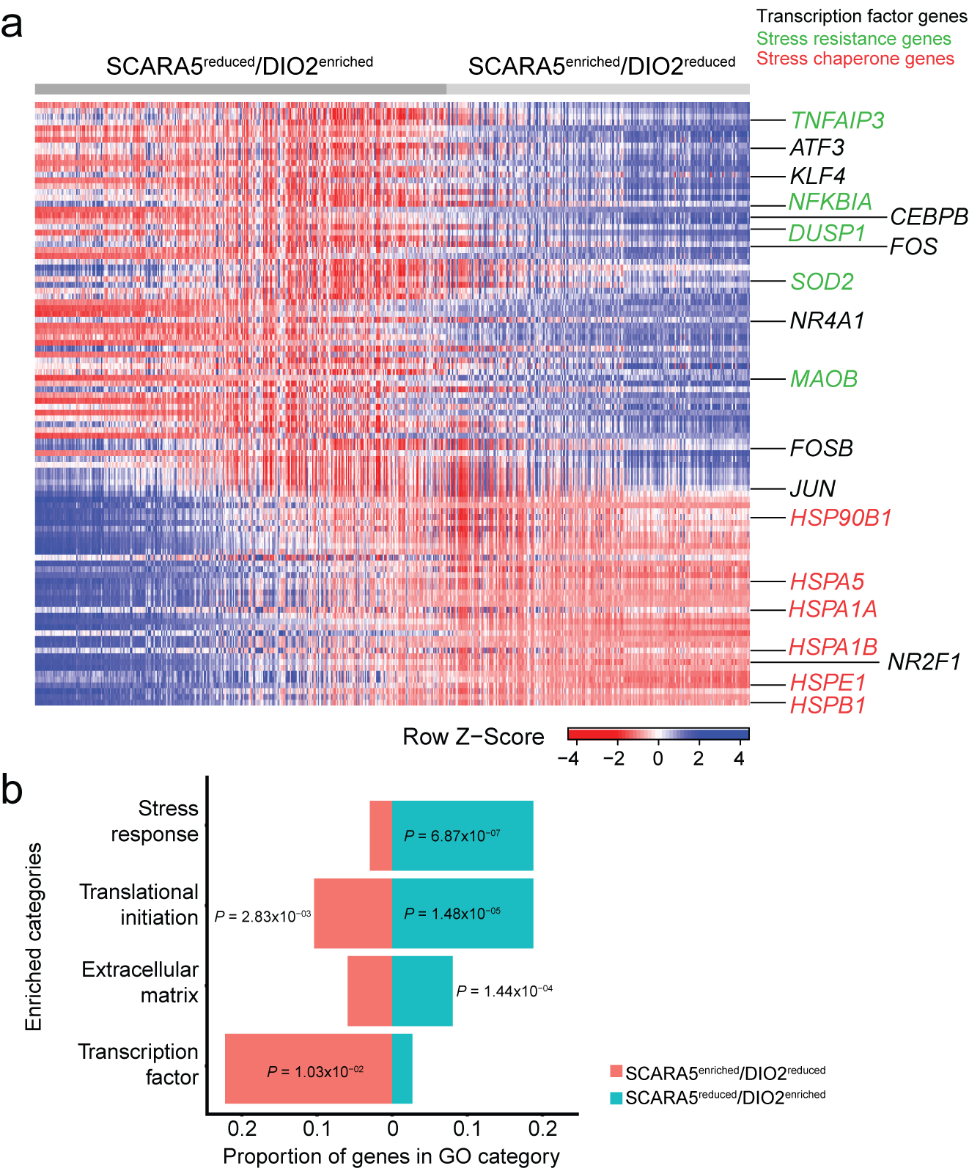


**Supplementary Figure 11. Differential gene expression in *SCARA5*^enriched^/*DIO2*^reduced^ EnSC versus *SCARA5*^reduced^/*DIO2*^enriched^ EnSC *in vivo*. a** Heatmap showing relative expression (z-score) of 104 DEG, based Wilcoxon rank sum test with Bonferroni corrected *P*-value < 0.05, between *SCARA5*^reduced^/*DIO2*^enriched^ EnSC (n=368) and *SCARA5*^enriched^/*DIO2*^reduced^ EnSC (n=271). Columns represent individual EnSC grouped according to their *SCARA5* and *DIO2* transcript levels. *SCARA5*^enriched^/*DIO2*^reduced^ EnSC were enriched in genes encoding several key decidual TF, including *CEBPB* ^12,13^, *NR4A1* (also known as *NUR77*) ^14^ and members of the AP-1gene family (*FOS*, *FOSB*, and *JUN*) ^14,15^. *SCARA5*^enriched^/*DIO2*^reduced^ EnSC were also enriched in genes involved in stress defences, including *SOD2* (coding superoxide dismutase 2) ^16^, *DUSP1* (coding MKP1) ^1^, *TNFAIP3* (TNF alpha induced protein 3), and *NFKBIA* (NFKB inhibitor alpha, IκBα) TNFAIP3 is a ubiquitin-modifying enzyme and potent inhibitor of NF-kappa B activation as well as TNF-mediated apoptosis. It plays a critical role in preventing inflammation *in vivo* ^17^. Likewise, IκBα potently suppresses inflammation by binding to NF-kappa B. By contrast, enhanced expression of genes coding various heat shock proteins (HSPs) in *SCARA5*^reduced^/*DIO2*^enriched^ cells is indicative of a proteotoxic stress response ^18^. **b** Gene ontology analysis revealed significantly enriched biological themes associated with *SCARA5*^reduced^*DIO2*^enriched^ versus *SCARA5*^enriched^/*DIO2*^reduced^ EnSC. The X-axis shows the proportion of differentially expressed genes found in each biological category. Benjamini-adjusted *P*-values (Fisher’s exact test) are shown.

**Supplementary Tables**

**Supplementary Table 1.** Contribution of individual samples to the endometrial cell populations in **a** *in vitro* timecourse and **b** *in vivo* biopsy analysis

**a**

| **Population** | **D0** | **D2** | **D4** | **D6** | **D8** | **WD** |
| --- | --- | --- | --- | --- | --- | --- |
| **S1** | 16 | 2 | 0 | 0 | 0 | 0 |
| **S2** | 692 | 3 | 1 | 1 | 0 | 14 |
| **S3** | 0 | 719 | 91 | 2 | 0 | 1 |
| **S4** | 0 | 44 | 567 | 52 | 13 | 3 |
| **S5** | 5 | 10 | 79 | 597 | 321 | 14 |
| **S6** | 4 | 2 | 2 | 102 | 351 | 104 |
| **S7** | 3 | 2 | 0 | 16 | 93 | 654 |

**b**

| **Population** | **P1** | **P2** | **P3** | **P4** | **P5** | **P6** |
| --- | --- | --- | --- | --- | --- | --- |
| **EC** | 6 | 30 | 8 | 37 | 36 | 24 |
| **EnSC** | 156 | 231 | 326 | 250 | 492 | 488 |
| **EpC1** | 3 | 90 | 47 | 15 | 27 | 11 |
| **EpC2** | 7 | 20 | 6 | 4 | 6 | 7 |
| **EpC3** | 0 | 1 | 66 | 2 | 0 | 0 |
| **EpC4** | 10 | 18 | 20 | 5 | 24 | 6 |
| **HP** | 0 | 1 | 0 | 3 | 5 | 7 |
| **IC1** | 3 | 3 | 1 | 0 | 0 | 3 |
| **IC2** | 2 | 0 | 1 | 3 | 5 | 5 |
| **IC3** | 6 | 6 | 0 | 0 | 0 | 0 |
| **NK1** | 8 | 18 | 16 | 2 | 17 | 14 |
| **NK2** | 14 | 28 | 47 | 2 | 12 | 29 |
| **NK3** | 51 | 16 | 22 | 1 | 3 | 14 |

|  |  |  |  |  |  |  |
| --- | --- | --- | --- | --- | --- | --- |

**Supplementary References**

1 Leitao, B. *et al.* Silencing of the JNK pathway maintains progesterone receptor activity in decidualizing human endometrial stromal cells exposed to oxidative stress signals. *FASEB journal : official publication of the Federation of American Societies for Experimental Biology* **24**, 1541-1551 (2010).

2 Leitao, B. B., Jones, M. C. & Brosens, J. J. The SUMO E3-ligase PIAS1 couples reactive oxygen species-dependent JNK activation to oxidative cell death. *FASEB J* **25**, 3416-3425, doi:10.1096/fj.11-186346 (2011).

3 Salker, M. S. *et al.* Deregulation of the serum- and glucocorticoid-inducible kinase SGK1 in the endometrium causes reproductive failure. *Nature medicine* **17**, 1509-1513, doi:10.1038/nm.2498 (2011).

4 Kajihara, T. *et al.* Differential expression of FOXO1 and FOXO3a confers resistance to oxidative cell death upon endometrial decidualization. *Mol Endocrinol* **20**, 2444-2455, doi:10.1210/me.2006-0118 (2006).

5 Muter, J. *et al.* Progesterone-Dependent Induction of Phospholipase C-Related Catalytically Inactive Protein 1 (PRIP-1) in Decidualizing Human Endometrial Stromal Cells. *Endocrinology* **157**, 2883-2893, doi:10.1210/en.2015-1914 (2016).

6 Kuroda, K. *et al.* Elevated periimplantation uterine natural killer cell density in human endometrium is associated with impaired corticosteroid signaling in decidualizing stromal cells. *The Journal of clinical endocrinology and metabolism* **98**, 4429-4437, doi:10.1210/jc.2013-1977 (2013).

7 Kuroda, K. *et al.* Induction of 11beta-HSD 1 and activation of distinct mineralocorticoid receptor- and glucocorticoid receptor-dependent gene networks in decidualizing human endometrial stromal cells. *Mol Endocrinol* **27**, 192-202, doi:10.1210/me.2012-1247 (2013).

8 Salker, M. S. *et al.* Disordered IL-33/ST2 activation in decidualizing stromal cells prolongs uterine receptivity in women with recurrent pregnancy loss. *PLoS One* **7**, e52252, doi:10.1371/journal.pone.0052252 (2012).

9 Al-Sabbagh, M. *et al.* NADPH oxidase-derived reactive oxygen species mediate decidualization of human endometrial stromal cells in response to cyclic AMP signaling. *Endocrinology* **152**, 730-740, doi:10.1210/en.2010-0899 (2011).

10 Weyemi, U. *et al.* ROS-generating NADPH oxidase NOX4 is a critical mediator in oncogenic H-Ras-induced DNA damage and subsequent senescence. *Oncogene* **31**, 1117-1129, doi:10.1038/onc.2011.327 (2012).

11 Uhlen, M. *et al.* Proteomics. Tissue-based map of the human proteome. *Science* **347**, 1260419, doi:10.1126/science.1260419 (2015).

12 Christian, M., Pohnke, Y., Kempf, R., Gellersen, B. & Brosens, J. J. Functional association of PR and CCAAT/enhancer-binding protein beta isoforms: promoter-dependent cooperation between PR-B and liver-enriched inhibitory protein, or liver-enriched activatory protein and PR-A in human endometrial stromal cells. *Mol Endocrinol* **16**, 141-154, doi:10.1210/mend.16.1.0763 (2002).

13 Christian, M. *et al.* Cyclic AMP-induced forkhead transcription factor, FKHR, cooperates with CCAAT/enhancer-binding protein beta in differentiating human endometrial stromal cells. *J Biol Chem* **277**, 20825-20832, doi:10.1074/jbc.M201018200 (2002).

14 Jiang, Y. *et al.* The orphan nuclear receptor Nur77 regulates decidual prolactin expression in human endometrial stromal cells. *Biochem Biophys Res Commun* **404**, 628-633, doi:10.1016/j.bbrc.2010.12.027 (2011).

15 Mazur, E. C. *et al.* Progesterone receptor transcriptome and cistrome in decidualized human endometrial stromal cells. *Endocrinology* **156**, 2239-2253, doi:10.1210/en.2014-1566 (2015).

16 Kajihara, T. *et al.* Human chorionic gonadotropin confers resistance to oxidative stress-induced apoptosis in decidualizing human endometrial stromal cells. *Fertil Steril* **95**, 1302-1307, doi:10.1016/j.fertnstert.2010.05.048 (2011).

17 Lee, E. G. *et al.* Failure to regulate TNF-induced NF-kappaB and cell death responses in A20-deficient mice. *Science* **289**, 2350-2354, doi:10.1126/science.289.5488.2350 (2000).

18 Nollen, E. A. & Morimoto, R. I. Chaperoning signaling pathways: molecular chaperones as stress-sensing 'heat shock' proteins. *J Cell Sci* **115**, 2809-2816 (2002).
