# Supplementary material for: Recurrent pregnancy loss is associated with a pro-senescent decidual response during the peri-implantation window
Source: Commun Biol. 2020 Jan 21;3:37. doi: 10.1038/s42003-020-0763-1 (PMC6972755; doi:10.1038/s42003-020-0763-1)
Supplement: Supplementary file 2 — Description of Additional Supplementary Files [file 42003_2020_763_MOESM2_ESM.pdf]

Description of Additional Supplementary Files

Supplementary Data (.xlsx file), containing:

Supplementary Data 1. Marker genes for each cell state *in vitro*

Supplementary Data 2. Differentially expressed genes (DEG) *in vitro* (S1-S7)

Supplementary Data 3. k-means cluster analysis (k=7)

Supplementary Data 4. GO analysis of co-expressed categories

Supplementary Data 5. Marker genes of endometrial cell types and subsets

Supplementary Data 6. Marker genes of immune cell clusters and sub-types

Supplementary Data 7. DEG in LH8 vs LH10 EnSCs

Supplementary Data 8. DEG in *SCARA5*^{enriched}/*DIO2*^{reduced} EnSC vs *SCARA5*^{reduced}/*DIO2*^{enriched} EnSC *in vivo*.
