# Supplementary material for: Recurrent pregnancy loss is associated with a pro-senescent decidual response during the peri-implantation window
Source: Commun Biol. 2020 Jan 21;3:37. doi: 10.1038/s42003-020-0763-1 (PMC6972755; doi:10.1038/s42003-020-0763-1)
Supplement: Supplementary file 4 — Reporting Summary [file 42003_2020_763_MOESM4_ESM.pdf]

nature research

Corresponding author(s): Jan Brosens

Last updated by author(s): Dec 17, 2019

Reporting Summary

Nature Research wishes to improve the reproducibility of the work that we publish. This form provides structure for consistency and transparency in reporting. For further information on Nature Research policies, see Authors & Referees and the Editorial Policy Checklist .

Statistics

For all statistical analyses, confirm that the following items are present in the figure legend, table legend, main text, or Methods section.

n/a	Confirmed	
☐	☒	The exact sample size (*n*) for each experimental group/condition, given as a discrete number and unit of measurement
☐	☒	A statement on whether measurements were taken from distinct samples or whether the same sample was measured repeatedly
☐	☒	The statistical test(s) used AND whether they are one- or two-sided<br>*Only common tests should be described solely by name; describe more complex techniques in the Methods section.*
☐	☒	A description of all covariates tested
☐	☒	A description of any assumptions or corrections, such as tests of normality and adjustment for multiple comparisons
☐	☒	A full description of the statistical parameters including central tendency (e.g. means) or other basic estimates (e.g. regression coefficient) AND variation (e.g. standard deviation) or associated estimates of uncertainty (e.g. confidence intervals)
☐	☒	For null hypothesis testing, the test statistic (e.g. *F*, *t*, *r*) with confidence intervals, effect sizes, degrees of freedom and *P* value noted<br>*Give P values as exact values whenever suitable.*
☒	☐	For Bayesian analysis, information on the choice of priors and Markov chain Monte Carlo settings
☒	☐	For hierarchical and complex designs, identification of the appropriate level for tests and full reporting of outcomes
☐	☒	Estimates of effect sizes (e.g. Cohen's *d*, Pearson's *r*), indicating how they were calculated

Our web collection on statistics for biologists contains articles on many of the points above.

Software and code

Policy information about availability of computer code

Data collection	Software used include: DropSeq Pipeline v1.0.1 and STAR v2.5.3a. Relevant parameters described in Methods section.
Data analysis	Software used: Seurat v2, Monocle v2.6.1, MeV v4.8, MAGIC, R v3.5 and GraphPad Prism v8. Relevant parameters described in Methods section.

For manuscripts utilizing custom algorithms or software that are central to the research but not yet described in published literature, software must be made available to editors/reviewers. We strongly encourage code deposition in a community repository (e.g. GitHub). See the Nature Research guidelines for submitting code & software for further information.

Data

Policy information about availability of data

All manuscripts must include a data availability statement. This statement should provide the following information, where applicable:
- Accession codes, unique identifiers, or web links for publicly available datasets
- A list of figures that have associated raw data
- A description of any restrictions on data availability

Data is available in GEO: www.ncbi.nlm.nih.gov/geo/ using the accession number GSE127918

Field-specific reporting

Please select the one below that is the best fit for your research. If you are not sure, read the appropriate sections before making your selection.

☒ Life sciences ☐ Behavioural & social sciences ☐ Ecological, evolutionary & environmental sciences

October 2018

Life sciences study design

All studies must disclose on these points even when the disclosure is negative.

Sample size	Power calculations were not performed to predetermine sample size. The number of biological repeat experiments is in line with common practice.
Data exclusions	One dataset was excluded from the single cell analysis of midluteal biopsies due to a considerable batch effect introduced by processing of this sample in isolation from the six reported in the manuscript.
Replication	Single-cell RNA-seq was validated on three independent human primary endometrial stromal cell cultures, and six endometrial biopsies in vivo. Four biological repeats were used for secretome analysis. In addition, 250 timed endometrial biopsies were used to generate the percentile graphs, and 90 and 89 biopsies of control subjects and recurrent pregnancy loss patients, respectively, were analyzed. The sample size of clinical samples was determined by the availability of biopsy samples from patients with the appropriate phenotype.
Randomization	No randomization was performed.
Blinding	Research staff were blinded to clinical phenotype

Reporting for specific materials, systems and methods

We require information from authors about some types of materials, experimental systems and methods used in many studies. Here, indicate whether each material, system or method listed is relevant to your study. If you are not sure if a list item applies to your research, read the appropriate section before selecting a response.

Materials & experimental systems

n/a	Involved in the study	
☒	☐	Antibodies
☒	☐	Eukaryotic cell lines
☒	☐	Palaeontology
☒	☐	Animals and other organisms
☐	☒	Human research participants
☒	☐	Clinical data

Methods

n/a	Involved in the study	
☒	☐	ChIP-seq
☒	☐	Flow cytometry
☒	☐	MRI-based neuroimaging

Human research participants

Policy information about studies involving human research participants

Population characteristics	Human endometrial biopsies were obtained from women attending the Implantation Research Clinic, a dedicated research clinic at University Hospitals Coventry and Warwickshire (UHCW) National Health Service Trust. Samples were obtained during the luteal phase of ovulatory, non-hormonally stimulated menstrual cycles. Overt uterine pathology was excluded by transvaginal ultrasound scan prior to the biopsy. Control subjects were women awaiting IVF treatment for a variety of reasons, including male-factor, unexplained, and tubo-ovarian infertility. Control subjects were considered to have good prognosis; patients with recurrent implantation failure, defined as 3 or more consecutive IVF failures with good quality embryos, were excluded. Recurrent pregnancy loss (RPL) patients had 3 or more consecutive miscarriages. Demographic details are presented in Table 1.
Recruitment	Subjects attending the Implantation Research Clinic at University Hospitals Coventry and Warwickshire (UHCW) National Health Service Trust.
Ethics oversight	Written informed consent was obtained from all participants in accordance with the guidelines in The Declaration of Helsinki 2000. The study was approved by the National Health Service National Research Ethics – Hammersmith, Queen Charlotte's & Chelsea Research Ethics Committee (1997/5065). For this study, surplus tissue was used from endometrial biopsies obtained for diagnostic purposes at the Implantation Research Clinic. All samples were stored in the Tommy's/Arden Biobank at UHCW. All human samples were treated in accordance with the regulations of the Human Tissue Authority and are compliant with the act. All persons handling human tissue are required to undertake training and maintain a biannual renewal of an institutional certificate to continue such work.

Note that full information on the approval of the study protocol must also be provided in the manuscript.
